# REAl world Dementia OUTcomes (READ-OUT) protocol: observational study

**DOI:** 10.1136/bmjopen-2025-115574

**Published:** 2026-06-25

**Authors:** David J Whiteside, Yi Yang, Ahmet Begde, Hannah Rome-Hall, Fatemeh Koohi, Ivan Koychev, Vanessa Raymont, James B Rowe, On behalf of the READ-OUT study group, Bernadette McGuiness

**Affiliations:** 1 Department of Clinical Neurosciences, University of Cambridge, Cambridge, UK; 2 Cambridge University Hospitals NHS Foundation Trust, Cambridge, UK; 3 Department of Psychiatry, Medical Sciences Division, University of Oxford, Oxford, UK; 4 Medical Research Council Cognition and Brain Sciences Unit, University of Cambridge, Cambridge, UK

**Keywords:** Dementia, Vulnerable Populations, Brain, Observational Study

## Abstract

**Abstract:**

**Introduction:**

Developments in blood biomarkers (BBMs) in dementia call for studies that evaluate their diagnostic, prognostic and societal implications in real-world populations. Such evidence is essential to support the safe and effective integration of BBMs into clinical practice.

**Methods and analysis:**

The REAl world Dementia OUTcomes study (READ-OUT) is a UK-based, multi-site study that assesses the prognostic and diagnostic utility of BBMs in people attending memory assessment services (‘secondary care’). READ-OUT phase 1 is a 3-year observational study of 3165 people representative of the UK population, with up-sampling of under-represented groups and proactive recruitment to include individuals missing from secondary care memory services. Participants are over the age of 45, able to provide a blood sample and willing to consent to linkage with electronic health records. The baseline study visit includes venepuncture and questionnaire assessment of quality of life and health resource usage. A subsample of the full cohort will have further venepuncture and questionnaires at a 52-week follow-up. READ-OUT substudies will evaluate BBMs’ test–retest reliability, the impact of processing delays and the feasibility of self-collected blood-spot samples. The clinical utility of BBMs will be assessed against diagnosis, outcomes from electronic health records, corollary data and health economic data. Results of the observational study discussed in this protocol will inform the design of a randomised controlled trial to test the cost-effectiveness, acceptability and clinical utility of disclosed BBM results.

**Ethics and dissemination:**

The study has received Research Ethics Committee approval (REC reference 24/WA/0330) and approval through the Health Research Authority/Health and Care Research Wales (IRAS project 342412). Anonymised study data will be stored on the Dementia Platform UK (DPUK) Data Portal (https://portal.dementiasplatform.uk) and made available through a managed access process, subject to the DPUK Data Access Agreement.

**STRENGTHS AND LIMITATIONS OF THIS STUDY**

- REAl world Dementia OUTcomes (READ-OUT) is a multi-centre real-world cohort of the diagnostic and prognostic utility of blood-based biomarkers in N=3165 people with cognitive and behavioural symptoms.
- This study evaluates blood biomarkers for dementia in a cohort that reflects the diversity of the UK population.
- Thresholds for optimal biomarker performance will be informed by patient/carer input.
- Results of READ-OUT’s observational study will determine the second study phase, a randomised controlled trial of biomarker disclosure.
- Diagnostic confirmation will draw on expert review of notes, longitudinal follow-up, with gold standard genetics, positron emission tomography and neuropathology in a subset of patients.

## Introduction

 The number of people living with dementia and neurodegenerative disease is rising in the UK and globally,^1 2^ with costs for the individual, their families and carers, and wider society.^3^,^5^ There is a pressing need for accurate and early diagnosis of dementias, supporting clinical pathways and anticipated disease-modifying treatments that are specific to the underlying neuropathology. Early and accurate diagnosis allows instigation of effective interventions and support at a point when a person with dementia is able to make decisions about their care,^5^ and reducing the anxiety and carer burden arising from diagnostic uncertainty and delay.^46^,^8^ The management of dementia is evolving, driven by positive phase three trials of disease-modifying therapies for Alzheimer’s disease (AD),^9 10^ which require confirmation of amyloid positivity.

The current diagnosis of dementia in memory clinics typically rests on a combination of clinical assessment, neuropsychological testing and neuroimaging by MRI or CT.^11^ Clinical and cognitive assessments can be inconclusive or incorrect even at expert memory centres,^12^ while standard structural imaging and clinical biomarkers lack molecular specificity even where the clinical diagnosis is correct. A small proportion of patients at UK memory centres have access to pathologically specific biomarker testing through positron emission tomography (PET) imaging or lumbar puncture, but these are resource-intensive and not widely available.^13 14^

Recent developments have highlighted the potential use of blood tests reflecting the underlying pathology of dementia, particularly the beta-amyloid burden in AD.^15^,^22^ In research cohorts, they have been shown to accurately differentiate individuals with AD when compared with clinical diagnoses,^17^ gold standard PET or cerebrospinal fluid (CSF) biomarkers^23 24^ and amyloid neuropathology.^25^ Blood biomarkers (BBMs) have also been shown to have prognostic value both in people with dementia and in at-risk cohorts.^1626^,^28^ Novel CSF biomarker developments may enable identification of non-amyloid pathology in neurodegenerative disease,^29^ but BBMs of these pathologies are not yet validated for clinical practice.

Although BBMs have the potential to improve diagnostic accuracy and prediction of outcomes for people with dementia, they are not yet integrated into standard practice within health services, including the UK National Health Service (NHS). Research study cohorts are younger, less comorbid and less ethnically diverse than patients in real-world memory services^30^ and so further work is needed to establish the utility and efficacy of such tests in well-powered real-world (‘minimally selected’) populations.^31 32^ In addition, knowledge about the impact on biomarker-based diagnosis for people with dementia is incomplete, including our understanding of whether people want to know the results of imperfect tests, what they want to know, and how this information is best conveyed.^33^,^35^

The REAl world Dementia Outcomes (READ-OUT) study assesses the diagnostic and prognostic utility of BBMs for people attending memory clinics, in a participant group representative of the diversity of people with dementia or cognitive symptoms in the UK. The study is divided into two consecutive phases, with phase 1 consisting of a 3-year observational study of 3165 people, followed by a randomised controlled trial of disclosure (phase 2). Recruitment for READ-OUT builds on the Dementia Platform UK (DPUK) Trial Delivery Framework (TDF), a network of NHS hospital services, healthcare commissioners, higher education institutions and NHS primary care networks. This network enables rapid, cost-effective recruitment of accurately characterised individuals to assess BBMs.

BBMs are considered in three *Tiers* determined by the current research evidence or clinical practice: tier 1 tests have either phase 3 clinical evidence, meeting standards set out by the Alzheimer’s Association,^36^ or are in current healthcare use; tier 2 tests have extensive research evidence but without evidence meeting criteria for tier 1; and tier 3 includes other, novel or exploratory biomarkers. READ-OUT will initially test tier 1 and tier 2 biomarkers, and retain serum, plasma and DNA (without cells) for tier 3 BBM work in partnership with academic and industry groups. This protocol focuses on phase 1 of the study, with a further protocol to be published detailing the second study phase. The research questions of the combined study are set out in the following section.

### Summary of research questions

READ-OUT is designed to test hypotheses associated with five central research questions across the observational study arm (READ-OUT phase 1) and randomised controlled trial (READ-OUT phase 2). Research questions 1–3 will be addressed in the observational study arm, while questions 4–5 will require data from the combined READ-OUT phase 1 and phase 2 studies.

1. What is the utility of BBMs for people in memory assessment services?
  - Which BBMs are needed to test the hypothesis that BBMs are cost-effective and ready for healthcare application?
  - How do BBMs perform in terms of their:
    - Diagnostic utility for the accuracy of detection of AD and non-Alzheimer’s dementias?
    - Prognostic utility?
    - Variance arising from characteristics of individual participants, including sex, age, multimorbidities, ethnicity, level of deprivation?
    - Utility within clinical pathways from the viewpoints of stakeholders?
2. Which BBMs give optimal performance?
  - Which biomarker or set of biomarkers is useful for dementia diagnosis and prognostication?
  - How do BBMs perform when considering complementary validations, against:
    - Clinical assessment, including the use of brain imaging.
    - Long-term electronic health record linkage.
    - Selected case reviews by independent experts, against disease-specific consensus criteria.
    - Comparison with genetics, CSF biomarkers and amyloid-PET imaging.
3. What mode of blood-based biomarker assessment gives optimal performance in real-world populations? READ-OUT considers the challenges to biomarker service delivery, including whether:
  - BBMs are robust to processing delays?
  - Phlebotomy can be substituted by remote blood test kits, such as blood-spot cards, including where these are posted to or from participants?
  - Test–retest reliability is sufficiently high for management decisions for individual patients?
4. What are the effectiveness and cost-effectiveness of BBMs? READ-OUT assesses the intended applications of BBMs in clinical practice in terms of:
  - Biomarker impact on health outcomes.
  - Total lifetime healthcare costs.
  - Quality-adjusted life years.
  - Cost-effectiveness and impact on budgets, including impact on affordability, implementation in clinical practice and variance depending on timing in the clinical pathway.
  - Quantitative and qualitative evaluations of blood-based biomarker testing and disclosure, to understand what information patients want and the communication of risk and effectiveness.
5. How do we ensure a blood-based biomarker pathway that is scalable and sustainable?
  - What is required for a sustainable, scalable national health services model for BBMs?
  - How can BBMs be delivered across the UK?

## Methods and analysis

### Study design

**Figure 1:**
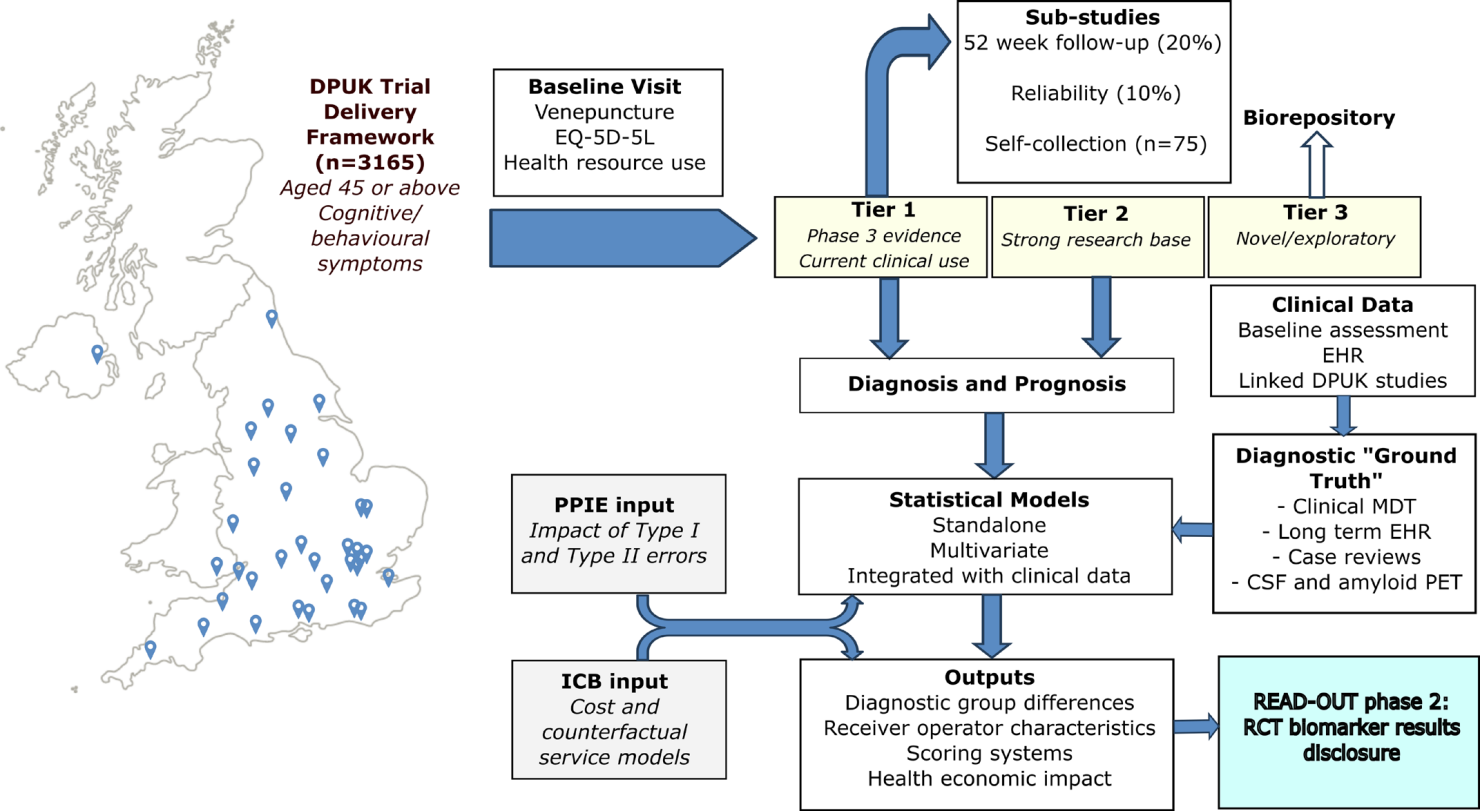
Schematic of the study design for REAl world Dementia OUTcomes (READ-OUT) phase 1. Participants (N=3165) are recruited from centres in the Dementia Platform UK (DPUK) Trial Delivery Framework. Blood-based biomarkers are considered in three tiers depending on research evidence and current clinical use. Diagnostic and prognostic utility will be assessed with a range of statistical methods, including incorporating linked clinical data. Thresholds will be determined with feedback from the patient and participant involvement and engagement group, and health economic impact using information from Integrated Care Boards (ICBs). Results of the observational arm of READ-OUT will feed into a randomised controlled trial (RCT) of blood-biomarker results disclosure. CSF, cerebrospinal fluid; EHR, electronic health record; MDT, multidisciplinary team; PET, positron emission tomography; PPIE, public and patient involvement and engagement.

The READ-OUT study protocol consists of two consecutive phases designed to address the research hypotheses. The first phase (figure 1) is a 3-year observational-design study in 3165 people using a broad panel of BBMs, with a 52-week follow-up in 20% of participants. The study started on 1 November 2024. The study baseline visit includes blood sampling, self-reported instruments to assess health and health resource usage, and collation of demographic details and medical history. Substudies will investigate the effect of sample processing delays, test–retest reliability of BBMs and the utility of blood spot cards. In the third year of the study, a panel of experts, including people with lived experience of dementia and their carers, will review the evidence from the observational study to determine BBM choices and generate clinician and patient-facing materials detailing the significance of the BBM results.

Phase 2 of READ-OUT is a randomised controlled trial to test the cost-effectiveness, acceptability and clinical utility of disclosed BBMs to patients and their clinicians. The final protocol for READ-OUT phase 2 will be set out in detail in a further publication.

### Participant recruitment and selection

The observational phase of READ-OUT will recruit 3165 participants. Participants are over the age of 45, able to provide blood samples and willing to consent to linkage to NHS health records (box 1). There are no restrictions on diagnosis or severity. People with cognitive symptoms attending secondary care services for dementia and related conditions (‘memory clinics’) are eligible, regardless of the diagnosis emerging from assessment in those services. Participants must provide informed consent, unless the participant does not have capacity for consent to study participation, in which case advice will be sought from a consultee or their guardian/attorney as set out in the relevant legislation in the devolved nations of the UK.

Box 1 **Inclusion and exclusion criteria for READ-OUT phase 1**

**Inclusion criteria**

Aged 45 or above.

Referred or referable with cognitive or behavioural symptoms and/or diagnosed with a cognition-related disorder; including those with subjective cognitive impairment and functional disorders.

Willing and able to give informed consent for participation in the study.

OR

Adults who otherwise lack the capacity to consent, but for whom advice regarding participation has been obtained via a consultee using the personal consultee process.

**Exclusion criteria**

Lack of venous access.

Unwilling to consent to NHS data linkage.

NHS, National Health Service; READ-OUT, REAl world Dementia OUTcomes.

90% of participants will be recruited from memory clinics at selected clinical sites across the UK embedded within the DPUK TDF. Participants will be approached by their clinical team, or by affiliated researchers, where they are part of a relevant research registry or cohort. Contact information for interested participants will be forwarded to the site study team. The further 10% of participants will not currently be in secondary care services but would be eligible for referral to secondary care memory services. This group is loosely defined, in order to account for the under-representation of certain communities and individuals from regions of higher socioeconomic deprivation in current UK memory services, who in other contexts would be expected to be in contact with secondary care services for their symptoms. Participants will be identified by primary care providers based at DPUK TDF sites who will search for individuals who have either evidence for subjective or objective cognitive difficulties, or a history of recurrent delirium, and no recorded diagnosis of a cognitive disorder. Primary care providers will contact the participant and will pass on contact details to the local study team with the participant’s permission. Following the study visit, relevant information will be passed on to the participant’s primary care provider with their agreement for consideration of further work-up or referral as needed.

READ-OUT aims for 30% recruitment in traditionally underserved and under-represented groups based on ethnicity, comorbidities and socioeconomic deprivation. To achieve this aim, we use census and community mapping to prioritise recruitment at DPUK TDF and DPUK Partner Akrivia Health sites, and from DPUK’s Feasibility and Acceptability of Scalable Tests of brain health study and ON-FIRE frontotemporal dementia study. We will prioritise sites with high levels of under-represented populations, using the NIHR-INCLUDE guidelines and NIHR-INCLUDE Ethnicity Framework.^37^ This will be supported by a range of outreach techniques, including the use of community connectors, telemedicine, education around brain health, research buses aligned to memory services and the development of language and culturally specific study information.

### Sample size and power

Given that the assessment of clinical utility of BBMs requires accuracy, READ-OUT prioritises receiver operator characteristics. Diagnostic and prognostic performance metrics are accuracy, sensitivity, specificity and positive- and negative-predictive values. A cohort size of N~3000 allows comparison of tests in subgroups with multiple predictors to detect events (dementia diagnosis, dementia aetiology, prognostication) and greater than 10 events per predictor using unbiased logistic regression, machine learning approaches, and out-of-sample testing.^38 39^ Power for individual tests will vary according to the prevalence of each dementia type in the cohort. Indicative frequentist power calculations were performed in R V.4.4.1 using the *powerMediation*^40^ packages. For example, we assume rates of mild cognitive impairment (MCI) of 17% in line with UK memory clinic^41^ with annual conversion rates from MCI to dementia of 10%–15%.^42 43^ A cohort of N=3165 gives 80% power at 5% significance to detect a change in the probability of converting to dementia within a year from 10% to 15% (an OR of 1.5) when the BBM is increased by 1 SD above the mean.

### Study procedures

#### Informed consent

Eligible individuals who express interest in participating will receive both verbal and written information about the READ-OUT study. This will include a clear statement that participation is voluntary and that individuals may withdraw at any time without affecting their current or future care, without compromising their legal rights and without needing to provide a reason.

Participants will be given time to review the information and, if they wish, to ask questions, either to the research team, their primary care provider, or to an independent party. After this period, written informed consent will be obtained. This involves the participant signing and dating the consent form, along with the dated signature of the individual who provided the study information and obtained consent. Consent will be taken by a qualified and experienced member of the research team who has been authorised by the chief or principal investigator.

A copy of the signed consent form will be offered to the participant and placed in their medical records where appropriate. The original will be securely stored at the study site. For participants with the mental capacity to consent, participation in the study requires valid informed consent.

READ-OUT will commission professional translation services to meet the needs of specific sites, in different parts of the country. For those unable to write, a witness independent of the study team can provide written evidence of the participant’s verbal consent. Independent consent will be sought at the baseline visit for participation in the READ-OUT substudies, with separate written and verbal information provided with the relevant information.

If an identified potential participant in England, Wales or Northern Ireland lacks capacity to consent to study participation, then advice will be sought from an appropriate consultee in accordance with the Mental Capacity Act (England and Wales 2005, Northern Ireland 2016). The consultee will be asked to sign a written declaration that they understand and accept the role of consultee. If a potential participant lacks the capacity to consent in Scotland, then a Personal Legal Representative will be asked to provide advice to the study team. This can be either a Welfare Guardian/Welfare Attorney or Nearest Relative as set out in the Adults with Incapacity Act (Scotland) 2016. If a participant without mental capacity declines to participate, this expression of wishes will be respected.

#### Baseline visit

**Figure 2:**
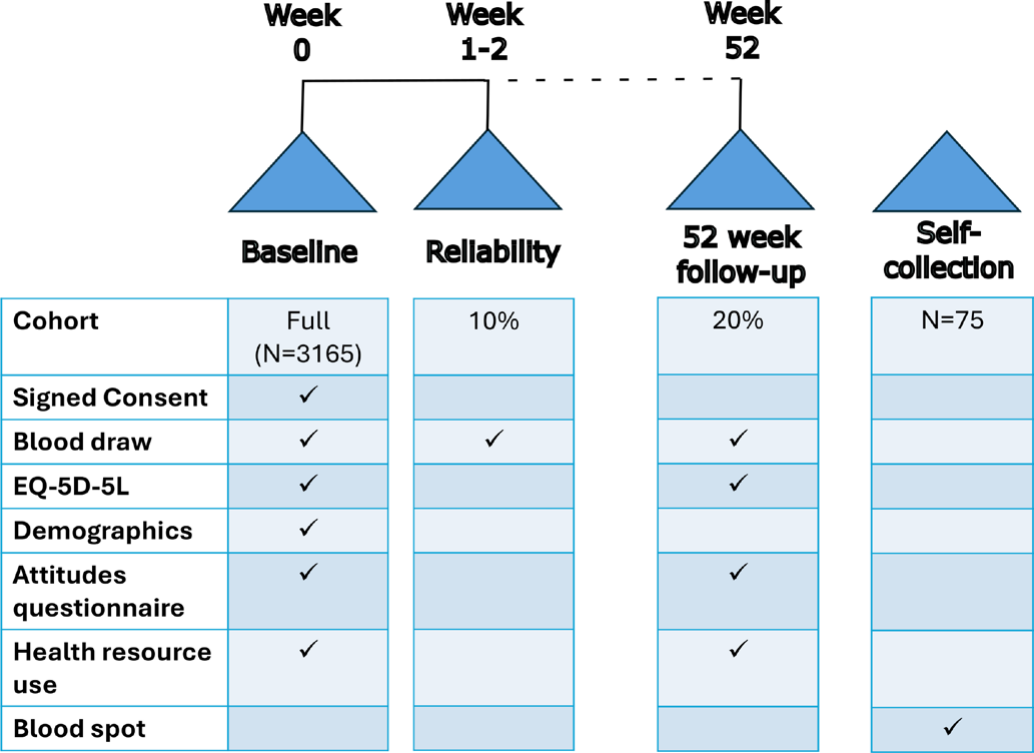
Schedule of events for the REAl world Dementia OUTcomes observational arm. EQ-5D-5L, EuroQol 5 Dimensions 5 Levels.

The schedule of events for the main READ-OUT study and substudies is set out in figure 2. The baseline study visit may occur on the same day as consent or after an interval if determined by patient choice or local circumstances. 40 mL of blood will be collected from participants via venepuncture prior to processing. Patient demographic information and details of the medical history will be obtained, as well as details of cognitive assessments and biomarker (CSF or PET) testing. Structured assessment will consist of a questionnaire about the participant’s attitude to blood-based biomarker testing for dementia, the EQ-5D-5L (EuroQol 5 Dimensions 5 Levels)^44^ instrument and a Health Resource Use Questionnaire.

#### READ-OUT studies

READ-OUT includes three optional substudies.

- Sample test–retest reliability and impact of delays in blood processing. 10% of the full cohort will be recruited to this substudy. Participants invited to this substudy will be invited to attend 1–2 weeks after the baseline visit. 24 mL of blood will be collected by venepuncture with sample processing as set out in the following section.
- Week 52 visit. This substudy will recruit 20% of the full cohort. Recruited participants will attend at 52 weeks for 20 mL blood sample collection. Participants will be asked to complete the Health Resource Use Questionnaire and the EQ-5D-5L instrument. The aim of the 52-week follow-up substudy is to support the design of the second phase of READ-OUT, including communication of BBM results and understanding biomarker variability, assisting with the estimation of cost-effectiveness and affordability, and determining optimal timing of BBM testing.
- Self-collection of blood through blood spot card testing. 75 participants will be recruited to this substudy. Participants who consent to take part in this substudy will be asked to provide a blood sample using a dried blood spot card. A prepaid envelope will be provided so the samples can be returned from home after collection. If needed, participants will receive both written instructions and a demonstration on how to collect the blood sample using the provided device.

#### Blood sampling and assay analysis

Blood sampling will be performed after consent. For most patients, 40 mL of blood will be obtained by venepuncture and collected in EDTA and SST tubes. In the main study, samples must be processed within 4 hours, though processing will occur as soon as possible. Samples will be centrifuged to separate plasma and serum at 2000 g for 10 min at 21 °C with no brake. All plasma and serum will be transferred to cryovials in specified volumes. Plasma will be divided into 4×250 µL, 4×500 µL and the remainder into 1 mL tubes. Serum will be divided into 4×250 µL, 2×500 µL and the remainder into 1 mL tubes. They will then be frozen at −80°C prior to transport to the central Biorepository, where they will be held until further analysis.

BBMs will be considered in three tiers:

Tier 1: this tier was set up to include BBMs currently in standard healthcare use, which in 2023 met criteria for phase 3 evidence performance in guidance from the Alzheimer’s Association, the precursor to current Clinical Practice Guidelines.^36^ These tests included phosphorylated tau (p-tau) 181, p-tau 217, neurofilament light chain, AB1-40/42 (with confirmatory testing ≥90% sensitivity and specificity in large and diverse clinical cohorts and/or clinical trials) and apolipoprotein E status.

Tier 2: BBMs were defined by substantial research cohort evidence, falling short of readiness for current healthcare use. These may reflect new assays of beta-amyloid pathology or other processes related to neurodegeneration, its risks or consequences. Assays include glial fibrillar acidic protein, chitinase-3-like protein 1 (YKL-40), progranulin, lipidomics, polygenic risk scores, without specifying the technology used to quantify them (funding restricted the initial phase of tier 2 analysis to Nucleic acid-Linked Immuno-Sandwich Assay Sequencing^45^ (NULISA), but alternative analysis platforms could also qualify for tier 2).

Tier 3: this tier focuses on novel and exploratory biomarkers, with samples retained in the biorepository for use by academic and industry partners. Such biomarkers might be metabolomic, proteomic, transcriptomic, genomic and epigenomic, targeted molecular assays or other biomarkers.

The samples will undergo genotyping using appropriate contemporary methods. Following quantification of extracted cell-free DNA on a Tapestation, whole-genome enzymatic methylation sequencing libraries will be prepared using standard protocols. Sequencing will be performed on the Illumina NovaSeq X+platform at a depth of 30–50× coverage, or a more advanced platform if available at the time of analysis. It is expected that genotyping methodologies may evolve over the course of the study.

Blood samples will be taken for the three subgroups to assess biomarker assay reliability and opportunities for service delivery. To evaluate assay test–retest reliability, participants will have a repeat venepuncture at 1–2 weeks post the baseline visit. At this second visit, four EDTA plasma tubes will be collected. One tube will be kept at room temperature for 2 hours prior to processing, a second tube at 3°C–5°C for 2 hours, a third tube at room temperature for 24 hours, and a fourth tube at 3°C–5°C for 24 hours. Subsequent processing will be as in the main study.

At the 52-week follow-up visit, blood sample processing will mirror the baseline visit.

#### Diagnostic confirmation

The diagnostic ‘ground truth’ will be established through four complementary routes:

1. Long-term follow-up. Evaluating the consistency of diagnoses over time, acknowledging that diagnostic accuracy improves with extended follow-up.
2. Consensus review. For a subset (10% of READ-OUT, up to 50% per main dementia group), a panel of three experienced clinicians (at consultant, clinical lecturer or senior lecturer level) will review records to provide a consensus clinical diagnosis.
3. Comparison with disease-specific gold-standard tests, including CSF biomarkers for Alzheimer’s, amyloid PET scans and genetic testing for known autosomal dominant causes of common dementias.
4. Neuropathology. Where available, findings from research studies or hospital/coroner neuropathological examinations will be used.

#### Data linkage and corollary data collection

For inclusion in the trial, participants consent to access to electronic health records via data linkage services provided by the NHS or equivalent. Data of interest includes information on therapeutics, inpatient hospital admissions, critical care admissions, attendance at outpatients and coded diagnoses. Following completion of the study, datasets derived from linkage will be held indefinitely if it retains research value to do so and we will seek appropriate ethical approvals to do so following the completion of this study. There is currently no equivalent data linkage service in Northern Ireland, and therefore in this region long-term follow-up will be managed by the local site team.

The READ-OUT dataset will include corollary data from participants where available from other studies. Permission will be obtained from the study participant and study team, examples to date include the Open Network for Frontotemporal dementia Inflammation REsearch, ON-FIRE (chief investigator Dr Maura Malpetti; variables: BBMs), Quantitative MRI in NHS Memory Clinic, QMIN-MC (chief investigator Timothy Rittman; variables: structural and functional MRI summary and clinical data), and the Deep and Frequent Phenotyping Study (chief investigator Vanessa Raymont; variables: CSF biomarkers, structural MRI summary and clinical data; neurophysiology).

### Data management and access

#### Participant research results

If a BBM like p-tau 217 is undertaken as part of a clinical healthcare pathway, the clinician can disclose the result as for any other clinical test. The results of the research-only tests will be pseudonymised and not routinely shared with individual participants. As the clinical relevance of the BBM results obtained for research purposes may be uncertain, these results will generally not be communicated to participants. While some BBMs may have an established role in clinical practice in specific scenarios, automatic disclosure of results to participants may have unexpected implications, noting background rates of p-tau 217 positivity in population cohorts.^46^ However, if a site’s clinical principal investigator believes that a specific result could help inform the clinical management of a participant, without directly disclosing the result to them, a request for access to that data may be made. Such requests will be reviewed and considered by the chief investigator or delegate on a case-by-case basis.

#### Participants confidentiality

The study will adhere to the General Data Protection Regulation (GDPR) and the Data Protection Act 2018, which require the de-identification of data as soon as practicable. The processing of participants’ personal data will be minimised. However, to enable data linkage with participants’ electronic medical records, it will be necessary to collect their full name, date of birth and NHS number. This information will be recorded on the baseline case report form (CRF), with pseudonymised data entered into a secured REDCap database,^47^ with identifiable fields held in a secure database with restricted access.

Each participant will be assigned a unique study identification number, which will be used in all study documents and electronic databases, except for the baseline CRF, where identifying details will be included. All paper records will be securely stored on-site and accessible only to authorised study staff. Limited access to records may be granted to authorised personnel for audit and monitoring purposes. Study staff will take all necessary measures to protect the confidentiality and privacy of participants’ personal data.

#### Data maintenance and access

Personal data collected for electronic health record linkage will be securely stored in a restricted-access database and accessed solely by authorised personnel, and only when necessary to complete NHS data linkage applications. Participant consent will be obtained prior to data collection. Pseudonymised READ-OUT clinical data, recorded during participant visits via CRFs, will be maintained in a separate restricted-access clinical database. Delegated site staff will have access only to their designated data access group, enabling them to enter participant data relevant to their site.

Anonymised READ-OUT data will be held on the DPUK Data Portal, or equivalent trusted research environment in the years ahead. The Data Portal is a secure remote access environment designed for the analysis of cohort data.^48^ A curated, standardised dataset will be made available to eligible researchers through the DPUK Trusted Research Environment (TRE), which forms the core of the DPUK Data Portal. The TRE offers secure, remote access to a wide range of statistical and analytical tools. It supports integration of diverse data types such as medical imaging, proteomics, digital device outputs, genetic data and electronic health records. The standardised dataset will include access to corollary data where participants are recruited to linked DPUK studies.

To protect data confidentiality, only analysis results may be exported; the anonymised READ-OUT dataset itself remains securely within the Data Portal. The TRE applies the highest data security standards (ISO 27001), suitable for NHS data linkage, and supports both anonymised access and advanced analytical methods, including single-site and federated learning.

Access to data is managed through a formal application and review process, with a median turnaround time of 22 days for eligible requests. More details on the access procedure can be found here: DPUK Data Portal.

### Statistical analysis of READ-OUT phase 1

#### BBM modelling and validation

The statistical analysis plan is designed to test the READ-OUT phase 1 research questions with tailored approaches incorporating different tiers of BBMs. Outcomes will be reported in line with relevant standards.^49^

For diagnostic modelling, both traditional statistical approaches (eg, logistic regression) and supervised or unsupervised machine learning techniques (eg, Random Forests,^50^ XGBoost,^51^ support vector machines) will be applied to develop and compare models for diagnostic classification. Performance will be evaluated for individual biomarkers, combinations of biomarkers and models integrating biomarkers with demographic and clinical information, accounting for variation across ethnicity and comorbidities. Diagnostic accuracy will be assessed using metrics such as sensitivity, specificity, positive and negative predictive values, and the area under the receiver operating characteristic curve. The NULISA and other platforms will output high-dimensional data, and we will consider internal validation and biomarker ranking, feature selection, and shrinkage and regularisation methods.

Optimal thresholding of model outputs will be informed by insights from the public and patient involvement and engagement (PPIE) arm of the study, recognising the real-world impact of false positives and false negatives.

Prognostic accuracy will be assessed using longitudinal data and linked electronic health records. Model selection will be guided by the data type and number of time points, and will include approaches such as change-from-baseline analysis of health-related quality of life at 1 year, multilevel and generalised additive models for repeated cognitive outcomes, and models that accommodate censored data for survival and longitudinal diagnostic changes. READ-OUT also leverages DPUK studies with gold-standard biomarker testing, including CSF and PET markers.

In relevant subgroups, we will also evaluate the reliability and resilience of the assays. This includes: (a) assessing deliverability and scalability by comparing standard phlebotomy (all participants) with alternative sampling methods, such as postal blood spot cards; (b) examining test–retest reliability using intraclass correlation coefficients, along with evaluating tolerance to processing delays; and (c) comparing performance across both mass spectrometry and other assay platforms.

#### Health economic assessment

Health economics data will be collected during the study to: (a) assess differences in costs and health-related quality of life based on prognosis risk identified by the BBMs and (b) support the development of a cost-effectiveness model for the early health technology assessment of BBM as a component of diagnosis.

Health-related quality of life will be measured using the EQ-5D-5L instrument at baseline and at the 52-week visit for 20% of the cohort. EQ-5D-5L responses will be converted into health utility values in accordance with National Institute for Health and Care Excellence guidance^52^ and combined with survival data to estimate quality-adjusted life years (QALYs). Participants’ perspectives on diagnostic and biomarker information will be explored through a qualitative question administered at study sites, involving 15–20 participants recruited both from this study and through the READ-OUT PPIE programme.

NHS secondary care resource use and medication data will be obtained through linkage with electronic health records and valued using national cost data. Use of primary care services, social care, productivity losses, informal care and other anticipated resource changes will be captured via self-reported questionnaires at baseline and at the 52-week visit (for the subsample), and similarly valued using national costs.

Differences in costs and EQ-5D-5L utility values by risk group at baseline and throughout the follow-up period will be analysed using generalised linear models. Associations between changes in BBM values (from baseline to the 52-week visit) and both costs and EQ-5D-5L utility values will be assessed using panel regression methods, including fixed and random effects models. Missing data will be handled in line with best practice for cost-effectiveness analyses.^53^ This READ-OUT phase 1 will assess the relationship between BBM results and baseline and longitudinal health-related quality of life assessments. The impact of BBM disclosure on these metrics will be assessed in a second clinical trial phase of READ-OUT.

We will develop economic models to evaluate the early cost-effectiveness of BBM and support their translation into clinical practice. NHS and societal perspectives will be considered, and the primary health outcome will be QALYs. The final economic model structure will be developed in collaboration with clinicians and epidemiologists involved in the study. Diagnostic performance metrics of BBMs will be incorporated directly into the model to link diagnostic accuracy with long-term outcomes and costs. Parameter estimates for the early health technology assessment models will be informed by data from READ-OUT phase 1 and supplemented with evidence from targeted and systematic literature reviews.

### Public and patient involvement and engagement

The design of READ-OUT was informed by a specialist PPIE group, including those with lived experience of dementia or of attendance at a memory clinic. A further PPIE group has been created to support the project through phases one and two, consisting of people who have attended secondary care memory clinics and individuals from under-represented groups thought to be at risk of dementia.

Viewpoints of the PPIE group will inform issues associated with the study research questions and will feed back to the statistical analysis plan and health economic analysis. A critical element of READ-OUT is understanding whether people want to know their results, what they want to know and how best this information is conveyed. We will use quantitative and qualitative data to design potential ways of conveying information, which we will feed back to our PPIE group to understand the best way of communicating the results of biomarkers and risk. These processes will be reviewed as we collect BBM data in under-represented groups, to ensure they remain as applicable and appropriate as possible.

### Current status

This study is active and currently recruiting (https://clinicaltrials.gov/study/NCT07238049).

## Ethics and dissemination

This study will be conducted in accordance with the principles of the Declaration of Helsinki. READ-OUT has received Research Ethics Committee approval (REC reference 24/WA/0330) and approval through the Health Research Authority/Health and Care Research Wales (IRAS project 342412).

Anonymised READ-OUT study data will be maintained on the Data Portal hosted by DPUK (https://portal.dementiasplatform.uk). Data will be made available with a managed access process through DPUK and subject to a DPUK Data Access Agreement.

Study investigators will publish manuscripts setting out real-world BBM performance in peer-reviewed publications. If multivariate biomarker sets improve performance, the READ-OUT team will develop a simplified scoring-/weighting- system to aid bedside utility. Aggregated study results will be communicated back to participants through newsletters, social media and updates on the study website.

## Supplementary material

10.1136/bmjopen-2025-115574 online supplemental file 1
