# Supplementary material for: REAl world Dementia OUTcomes (READ-OUT) protocol: observational study
Source: BMJ Open. 2026 Jun 25;16(6):e115574. doi: 10.1136/bmjopen-2025-115574 (PMC13311591; doi:10.1136/bmjopen-2025-115574)
Supplement: online supplemental file 1 [file bmjopen-16-6-s001.docx]

# **APPENDIX**

## **The READ-OUT study group**

Bernadette McGuiness, *Centre for Public Health, Institute of Clinical Sciences, Royal Victoria Hospital, Queen’s University Belfast, Belfast, UK*

Gosia Raczek*, Brighton and Sussex School of Medicine, Brighton, UK*

Elizabeth Coulthard, *Bristol Medical School, University of Bristol, Bristol, UK*

Benjamin Underwood, *Department of Psychiatry, University of Cambridge, Cambridge, UK*

Brian Tom, *Medical Research Council Biostatistics Unit, University of Cambridge, UK*

Timothy Rittman, *Department of Clinical Neurosciences, University of Cambridge, Cambridge, UK* and *Cambridge University Hospitals NHS Foundation Trust, Cambridge, UK*

Tom C. Russ, *University of Edinburgh and NHS Lothian, Edinburgh, UK*

Anne Corbett*, Medical School, University of Exeter, Exeter, UK*

Terence J. Quinn, *Institute of Cardiovascular and Medical Sciences, University of Glasgow, Glasgow, UK*

Emma Wolverson*, The Geller Institute of Ageing and Memory, University of West London, London, UK*

Rosie Dunn, *The Geller Institute of Ageing and Memory, University of West London, London, UK*

Chinedu Udeh-Momoh, *Neuroepidemiology and Ageing Research Unit, School of Public Health, Faculty of Medicine, The Imperial College of Science, Technology and Medicine, London, UK*

Paul Edison, *Division of Neurology, Department of Brain Sciences, Imperial College London, London, UK*

Dag Aarsland, *Department of Old Age Psychiatry, Institute of Psychiatry, Psychology and Neuroscienc, King's College London, London, UK*

Ross Dunne, *Geoffrey Jefferson Brain Research Centre, University of Manchester, Manchester, UK*

Chineze Ivenso, *Department of Psychiatry, Aneurin Bevan University Health Board, Newport, Wales*

Amanda Adler, *Radcliffe Department of Medicine, Medical Sciences Division, University of Oxford, Oxford, UK*

Clare E. Mackay, *Oxford Centre for Human Brain Activity, Wellcome Centre for Integrative Neuroimaging, Department of Psychiatry, University of Oxford, Oxford, UK*

Filipa Landerio, *Health Economics Research Centre, University of Oxford, Oxford, UK.*

John Gallacher, *Department of Psychiatry, University of Oxford, Oxford, UK*

Benjamin Tari, *Institute of Sport, Exercise and Health, University College London, London, UK*

Jose Leal, *Nuffield Department of Population Health, University of Oxford, Oxford, UK*

Sarah Bauermeister, *Department of Psychiatry, University of Oxford, Oxford, UK*

Sarah T. Pendlebury, *Wolfson Centre for Prevention of Stroke and Dementia, Wolfson Building, Nuffield Department of Clinical Neurosciences, John Radcliffe Hospital, University of Oxford, Oxford, UK; NIHR Oxford Biomedical Research Centre, Oxford University Hospitals NHS Foundation Trust, Oxford, UK; Departments of Acute General Internal Medicine and Geratology, Oxford University Hospitals NHS Foundation Trust, Oxford, UK.*

Simon Young*, Department of Psychiatry, University of Oxford, Oxford, UK and Dementia Platform UK*

Charles Marshall, *Centre for Preventive Neurology, Queen Mary University of London, London, UK*

Claudia Cooper, *Centre for Psychiatry and Mental Health, Wolfson Institute of Population Health, Queen Mary University London, London, UK*

Daniel J. Blackburn, *Department of Neurosciences, Sheffield Teaching Hospitals, NHS Foundation Trust, Royal Hallamshire Hospital, Sheffield, UK*

Jay Amin, *University of Southampton, Southampton, UK*

Atticus H. Hainsworth, *Molecular and Clinical Sciences Research Institute, St George’s University of London, UK, Department of Neurology, St George’s University Hospitals NHS Foundation Trust, London, UK*

Christopher Kipps, *University Hospital Southampton NHS Foundation Trust, Southampton, UK Clinical and Experimental Sciences, Faculty of Medicine, University of Southampton, Southampton, UK*

Dennis Chan, *Institute of Cognitive Neuroscience, University College London, London, UK*

Henrik Zetterberg, *Department of Psychiatry and Neurochemistry, The Sahlgrenska Academy at the University of Gothenburg, Mölndal, Sweden; Clinical Neurochemistry Laboratory, Sahlgrenska University Hospital, Gothenburg, Sweden; Department of Neurodegenerative Disease, UCL Queen Square Institute of Neurology, London, UK; UK Dementia Research Institute at UCL, London, UK; Hong Kong Center for Neurodegenerative Diseases, Hong Kong, China; UW Department of Medicine, School of Medicine and Public Health, Madison, WI, USA*
