# Supplementary material for: REAl world Dementia OUTcomes (READ-OUT) protocol: observational study
Source: BMJ Open. 2026 Jun 25;16(6):e115574. doi: 10.1136/bmjopen-2025-115574 (PMC13311591; doi:10.1136/bmjopen-2025-115574)
Supplement: Supplementary file 2 [file bmjopen-2025-115574.reviewer_comments.pdf]

PEER REVIEW HISTORY

BMJ Open publishes all reviews undertaken for accepted manuscripts. Reviewers are asked to complete a checklist review form and are provided with free text boxes to elaborate on their assessment. These free text comments are reproduced below.

ARTICLE DETAILS

Title (Provisional)

The Real World Dementia Outcomes (READ-OUT) protocol: Observational study

Authors

Whiteside, David J; Yang, Yi; Begde, Ahmet; Rome-Hall, Hannah; Koohi, Fatemeh; Koychev, Ivan; Raymont, Vanessa; Rowe, James B; ., READ-OUT study group

---

VERSION 1 - REVIEW

**Reviewer**	**1**
**Name**	**Gonzales, Mitzi M**
**Affiliation**	**UT Health Science Center San Antonio Official Bookstore**
**Date**	**24-Feb-2026**
**COI**	**None**

This manuscript provides an overview of the REAL world Dementia OUTcomes study (READ-OUT), which assesses the diagnostic and prognostic utility of ADRD blood-based biomarkers (BBMs) in memory clinic settings across the UK. The manuscript is well-written and most of the procedures are well-outlined. A few recommendations to strengthen the manuscript are provided below.

1. The first paragraph introduction states that FDA-approved disease modifying medications require detailed biomarker testing, but that is incorrect. They only require confirmation of amyloid positivity as well as MRI and APOE testing to ensure eligibility and safety.

2. In paragraph 3 of the introduction, the authors describe BBMs that reflect "the underlying pathology of dementia". It is important to be clear and note that while plasma p-tau 217 is a strong surrogate of amyloid pathology, there are not yet validated BBMs for other ADRD pathologies.

3. Paragraph 6 of the introduction loosely outlines the criteria for BBM tier staging; however, there is not enough detail to enable replication. The sensitivity and specificity or positive/negative predictive values for each Tier should be clearly outlined.

4. Please provide a rationale for conducting 12-month follow-up in only 20% of the cohort.

5. Please provide a rationale for withholding research results for biomarkers with clinical grade validation (e.g. p-tau 217, lipid panels)

6. Please address how health-related quality of life, as well as the downstream impact on care utilization, can be assessed if the BBM results are not disclosed.

VERSION 1 - AUTHOR RESPONSE

We thank the reviewer for their input which has improved the manuscript. We provide details of our adjustments to the paper and findings below in the point-by-point responses.

Reviewer: 1
Dr. Mitzi M Gonzales, UT Health Science Center San Antonio Official Bookstore

Comments to the Author:
This manuscript provides an overview of the REAL world Dementia OUTcomes study (READ-OUT), which assesses the diagnostic and prognostic utility of ADRD blood-based biomarkers (BBMs) in memory clinic settings across the UK. The manuscript is well-written and most of the procedures are well-outlined. A few recommendations to strengthen the manuscript are provided below.

REVIEWER: 1. The first paragraph introduction states that FDA-approved disease modifying medications require detailed biomarker testing, but that is incorrect. They only require confirmation of amyloid positivity as well as MRI and APOE testing to ensure eligibility and safety.

RESPONSE: We have adjusted the paragraph to state that disease-modifying medications in Alzheimer's disease requires confirmation of amyloid positivity. The original phrase referred to MRI, APOE and tests of amyloid positivity but clearly biomarker characterisation can be much more detailed and the revised section is more accurate.

REVIEWER: 2. In paragraph 3 of the introduction, the authors describe BBMs that reflect "the underlying pathology of dementia". It is important to be clear and note that while plasma p-tau 217 is a strong surrogate of amyloid pathology, there are not yet validated BBMs for other ADRD pathologies.

RESPONSE: We thank the reviewer for this suggestion. We have adjusted the paragraph in question to reflect this as follows:

Recent developments have highlighted the potential use of blood tests reflecting the underlying pathology of dementia, particularly the beta-amyloid burden in Alzheimer's disease.[15–22] In research cohorts, they have been shown to accurately differentiate individuals with Alzheimer's disease when compared to clinical diagnoses,[17] gold standard PET or cerebrospinal fluid (CSF) biomarkers,[23,24] and amyloid neuropathology. Blood biomarkers have also been shown to have prognostic value both in people with dementia and in at-risk cohorts.[16,27–29] Novel CSF biomarker developments may enable identification of non-amyloid pathology in

neurodegenerative disease,[29] but blood biomarkers of these pathologies are not yet validated for clinical practice.

REVIEWER: 3. Paragraph 6 of the introduction loosely outlines the criteria for BBM tier staging; however, there is not enough detail to enable replication. The sensitivity and specificity or positive/negative predictive values for each Tier should be clearly outlined.

RESPONSE: We have updated the operationalisation of our Tier 1-2-3 structure to provide greater clarity in page 11 of our manuscript. The Tier structure was not based directly on the sensitivity and specificity of the blood-based biomarker, but the level of evidence according to the Alzheimer Association classification given also the cohorts within which they have been tested. The evidential level for each Tier does correlate partly with the potential purpose, and aspect of the neurodegenerative cascade that they reflect. We include the following:

Tier 1: This tier was set up to include BBMs currently in standard healthcare use, which in 2023 met criteria for phase-3 evidence performance in guidance from the Alzheimer's Association, the precursor to current Clinical Practice Guidelines.[35] These tests included phosphorylated tau (p-tau) 181, p-tau 217, neurofilament light chain (NfL), AB1-40/42 (with confirmatory testing ≥90% sensitivity and specificity in large and diverse clinical cohorts and/or clinical trials) and APOE status.

Tier 2: BBMs were defined by substantial research cohort evidence, falling short of readiness for current healthcare use. These may reflect new assays of beta-amyloid pathology or other processes related to neurodegeneration, its risks or consequences. Assays include glial fibrillary acidic protein, chitinase-3-like protein 1 (YKL-40), progranulin, lipidomics, polygenic risk scores, without specifying the technology used to quantify them (funding restricted the initial phase of Tier 2 analysis to Nucleic acid-Linked Immuno-Sandwich Assay Sequencing but alternative analysis platforms could also quality for Tier 2).

Tier 3: This tier focuses on novel and exploratory biomarkers, with samples retained in the biorepository for use by academic and industry partners. Such biomarkers might be metabolomic, proteomic, transcriptomic, genomic and epigenomic, targeted molecular assays or other biomarkers.

REVIEWER: 4. Please provide a rationale for conducting 12-month follow-up in only 20% of the cohort.

RESPONSE: The principal aim of READOUT was to optimise *diagnostic* biomarkers, not biomarkers for longitudinal monitoring. Nonetheless, the study provided the opportunity for a subset of participants, without raising the burden on participants or overall costs excessively. In contrast to other current deep phenotyping studies of dementia (eg Biohermes, Biohermes2) READOUT sought to minimise participant burden so as to maximise inclusivity.

One aim of the 12-month follow-up sub-study is to support the design of a second phase of READ-OUT, including communication of BBM results, estimation of the proportion of participants who move from p-tau 217 negative or intermediate status to positive, and predicting annualised rates of change. The sub-study will provide sufficient information to determine these factors for the major diagnostic groups. We have updated our description of the READ-OUT sub-studies (page 10, second point under title '*READ-OUT Sub-studies*').

REVIEWER: 5. Please provide a rationale for withholding research results for biomarkers with clinical grade validation (e.g. p-tau 217, lipid panels)

RESPONSE: Some of the Tier 1 biomarkers are already entering clinical practice. If a BBM like p-tau 217 is undertaken as part of a clinical healthcare pathway, the clinician can disclose the result as for any other clinical test. The site PI may also request the p-tau 217 result if they believe that the result would improve the participant's clinical care.

However, there are important knowledge gaps about BBMs, even Tier 1 BBMs, that the research participants in READOUT can help to close. To consent a patient to a clinical BBM, and interpret the result properly, requires knowledge of the performance of the test in the clinical setting (accommodating the prior probabilities of each differential diagnosis), and with patients with co-morbidities, and non-white ethnicities. However, much of the evidence of BBMs has come from non-representative cohorts. Enough is known about BBMs to be concerned about potential effects of ethnicity and co-morbidity. In addition, the background rates of p-tau positivity in older adult populations suggest additional uncertainty as to the implications for automatic disclosure of results to READ-OUT participants (imagine for example the high rates of p-tau 217 in people >80 with non-AD dementias). We have updated the section titled 'Participant Research Results' to clarify this issue.

REVIEWER: 6. Please address how health-related quality of life, as well as the downstream impact on care utilization, can be assessed if the BBM results are not disclosed.

RESPONSE: READ-OUT phase 1 will test the accuracy and influences on BBM performance in diverse conditions, and the degree to which BBMs are associated with baseline and longitudinal health-related quality of life assessments. READ-OUT phase 2 will test how BBM result disclosure impacts on how health-related quality of life and healthcare costs. The READ-OUT phase 2 is beyond the scope of this current protocol, as the study design can not be determined until phase 1 results are known.

Associations between changes in BBM values (from baseline to the 52-week visit) and both costs and EQ-5D-5L utility values will be assessed using panel regression methods, including fixed and random effects models. Missing data will be handled in line with best practice for cost-effectiveness analyses.[52] This READ-OUT phase 1 will assess the relationship between BBM results and baseline and longitudinal health-related quality of life assessments. The impact of BBM disclosure on these metrics will be assessed in a second, clinical trial, phase of READ-OUT.

VERSION 2 - REVIEW

**Reviewer**	**1**
**Name**	**Gonzales, Mitzi M**
**Affiliation**	**UT Health Science Center San Antonio Official Bookstore**
**Date**	**30-Apr-2026**

COI

The authors have sufficiently addressed my critiques.
